# Control of white mold (*Sclerotinia sclerotiorum*) through plant-mediated RNA interference

**DOI:** 10.1038/s41598-023-33335-4

**Published:** 2023-04-20

**Authors:** Philip L. Walker, Dylan J. Ziegler, Shayna Giesbrecht, Austein McLoughlin, Joey Wan, Deirdre Khan, Vanessa Hoi, Steve Whyard, Mark F. Belmonte

**Affiliations:** Department of Biological Sciences, University of Manitoba, Winnipeg, MB R3T 2N2 Canada

**Keywords:** Biotechnology, Plant sciences

## Abstract

The causative agent of white mold, *Sclerotinia sclerotiorum*, is capable of infecting over 600 plant species and is responsible for significant crop losses across the globe. Control is currently dependent on broad-spectrum chemical agents that can negatively impact the agroecological environment, presenting a need to develop alternative control measures. In this study, we developed transgenic *Arabidopsis thaliana* (AT1703) expressing hairpin (hp)RNA to silence *S. sclerotiorum ABHYDROLASE-3* and slow infection through host induced gene silencing (HIGS). Leaf infection assays show reduced *S. sclerotiorum* lesion size, fungal load, and *ABHYDROLASE-3* transcript abundance in AT1703 compared to wild-type Col-0. To better understand how HIGS influences host–pathogen interactions, we performed global RNA sequencing on AT1703 and wild-type Col-0 directly at the site of *S. sclerotiorum* infection. RNA sequencing data reveals enrichment of the salicylic acid (SA)-mediated systemic acquired resistance (SAR) pathway, as well as transcription factors predicted to regulate plant immunity. Using RT-qPCR, we identified predicted interacting partners of *ABHYDROLASE-3* in the polyamine synthesis pathway of *S. sclerotiorum* that demonstrate co-reduction with *ABHYDROLASE-3* transcript levels during infection. Together, these results demonstrate the utility of HIGS technology in slowing *S. sclerotiorum* infection and provide insight into the role of *ABHYDROLASE-3* in the *A. thaliana*–*S. sclerotiorum* pathosystem.

## Introduction

Host-induced gene silencing (HIGS) takes advantage of the RNA interference (RNAi) pathway, which is conserved within most eukaryotic organisms^1,2^. This pathway can be triggered by dsRNA molecules recognized and further processed into small interfering RNAs (siRNAs) by Dicer-like proteins (DCLs). These siRNAs with sequence complementarity to target pathogen transcripts are subsequently bound to Argonaute proteins (AGOs), forming the RNA induced silencing complex (RISC) with complementary siRNAs guiding the RISC to target mRNA transcripts for degradation^3,4^. HIGS uses complementary transgene-expressed hairpin (hp)RNA that is capable of targeting a single gene’s transcripts, thus removing or reducing the risk of off-target effects on the agroecological environment^5^. Applications of HIGS have demonstrated success against a diverse set of fungal pathogens, including ascomycetes *Botrytis cinerea*^6^, *Fusarium graminearum*^7^ and *Verticillium dahlia*^8^, basidiomycetes *Ustilago hordei*^9^ and *Melampsora lini*^10^*,* and oomycetes *Phytophthora infestans*^11^ and *Hyaloperonospora parasitica*^12^. Successful HIGS-mediated control of a phytopathogen is dependent on choosing a target gene whose transcript abundance can be adequately reduced by the hairpin (hp)RNA-expressing transgene^13,14^. In prior studies, we explored the efficacy of the putative tRNA synthetase *ABHYDROLASE-3* as an RNAi candidate gene through the topical application of long dsRNA in spray-induced gene silencing (SIGS) to reduce pathogenicity of *S. sclerotiorium* on *B. napus* and *Arabidopsis thaliana*^15^. Significant reductions in *S. sclerotiorum* lesion progression post application of *ABHYDROLASE-3* targeting dsRNA molecules were correlated with reductions in target transcript levels as early as 24 h post infection. Subsequently, Wytinck et al.^16^ engineered *B. napus* to express hpRNAs targeting the *S. sclerotiorum ABHYDROLASE-3* through HIGS and demonstrated reduced disease severity in inoculated stem tissues, coupled with significant *ABHYDROLASE-3* transcript reduction. As *S. sclerotiorum* infection commonly initiates in leaves prior to progressing to stem tissues^17,18^, understanding how *ABHYDROLASE-3* silencing influences the host response directly at the site of infection can provide insight into the control *S. sclerotiorum* prior to systemic infection. Further, while *ABHYDROLASE-3* silencing has demonstrated reduced disease severity against *S. sclerotiorum* using SIGS and HIGS technology, its role in pathogenesis is still largely unknown and identification of interacting partners and pathways may provide new candidates to target through RNAi.

Plants activate innate immunity pathways in response to pathogen attack and are dependent on the recognition of specific pathogen-derived molecules^19^. These pathways are commonly separated into necrotrophic and biotrophic responses and demonstrate distinct modes of pathogen detection. For example, necrotrophic pathogens are detected through the presence of pathogen and damage associated molecular patterns (PAMPs/DAMPs) via pattern recognition receptors (PRRs) in pattern-triggered immunity (PTI), while in contrast, biotrophic pathogens are detected via intracellular NBS-LRR proteins detecting secreted effector molecules in effector-triggered immunity (ETI)^19^. Activation of these recognition pathways induces specific defense hormones which can further activate down-stream defense processes to appropriately respond to pathogen attack. These hormones include jasmonic acid (JA) and ethylene (ET) which can directly activate the induced systemic resistance (ISR) pathway and are associated with promoting physical barriers against necrotrophic pathogens^20^. Other phytohormones like salicylic acid (SA) activate the systemic acquired resistance pathway (SAR) leading to a local hypersensitive response as a defense against biotrophic infection^21,22^. While *S. sclerotiorum* has been previously categorized as a necrotrophic fungal pathogen, recent reports provide evidence of an early biotrophic phase^23,24^. *S. sclerotiorum* secretes pathogenicity factors including oxalic acid (OA) and a suite of cell wall degrading enzymes to weaken and break down host cells upon infection^25,26^. During the initial stages of infection, a brief biotrophic phase may occur, where OA and other secreted pathogenicity factors act to suppress host defenses, specifically the SA-mediated SAR pathway^23,24^. The inability of *S. sclerotiorum* to successfully suppress SAR may lead to increased resistance against *S. sclerotiorum* infection. Further support for this initial biotrophic phase comes from the identification of secreted effector molecules essential for pathogenicity^27^ and intracellular NBS-LRRs necessary for successful defense against *S. sclerotiorum*^28^. Lastly, synergistic effects between SA-mediated SAR and JA/ET-mediated ISR have been described against necrotrophic infection^29^ and that removal of either of these defense hormones will result in an unsuccessful defense response^30^*.*

In the current study, we engineered *A. thaliana* expressing hpRNA complementary to *S. sclerotiorum ABHYDROLASE-3* under the activity of a CaMV 35S promoter (AT35S::SS1G_01703RNAi herein referred to as AT1703). AT1703 *A. thaliana* was more tolerant to *S. sclerotiorum* infection with reduced lesion size, fungal load and *ABHYDROLASE-3* transcript abundance compared to wild-type Col-0. Light microscopy of the infection process further identified reduced epidermal and mesophyll tissue degradation and vascular tissue colonization in AT1703 lines. Global RNA sequencing revealed enrichment of the SA-mediated SAR pathway and predictive gene regulatory network analysis predicted heat shock factor (*HSFA4A*, *HSFA8*) and TGA (*TGA10*) transcription factors as putative regulators of resistance in AT1703. Analysis of *ABHYDROLASE-3* predicted interacting partners suggests this pathogenicity factor plays a role in the methionine salvage pathway which is necessary for successful polyamine biosynthesis. Taken together, these data demonstrate the utility of HIGS in slowing *S. sclerotiorum* infection and highlight the importance of the SA-mediated SAR pathway in contributing to successful defense against *S. sclerotiorum*.

## Results

### HIGS of *S. sclerotiorum ABHYDROLASE-3* slows infection in transgenic AT1703 Arabidopsis

**Figure 1:**
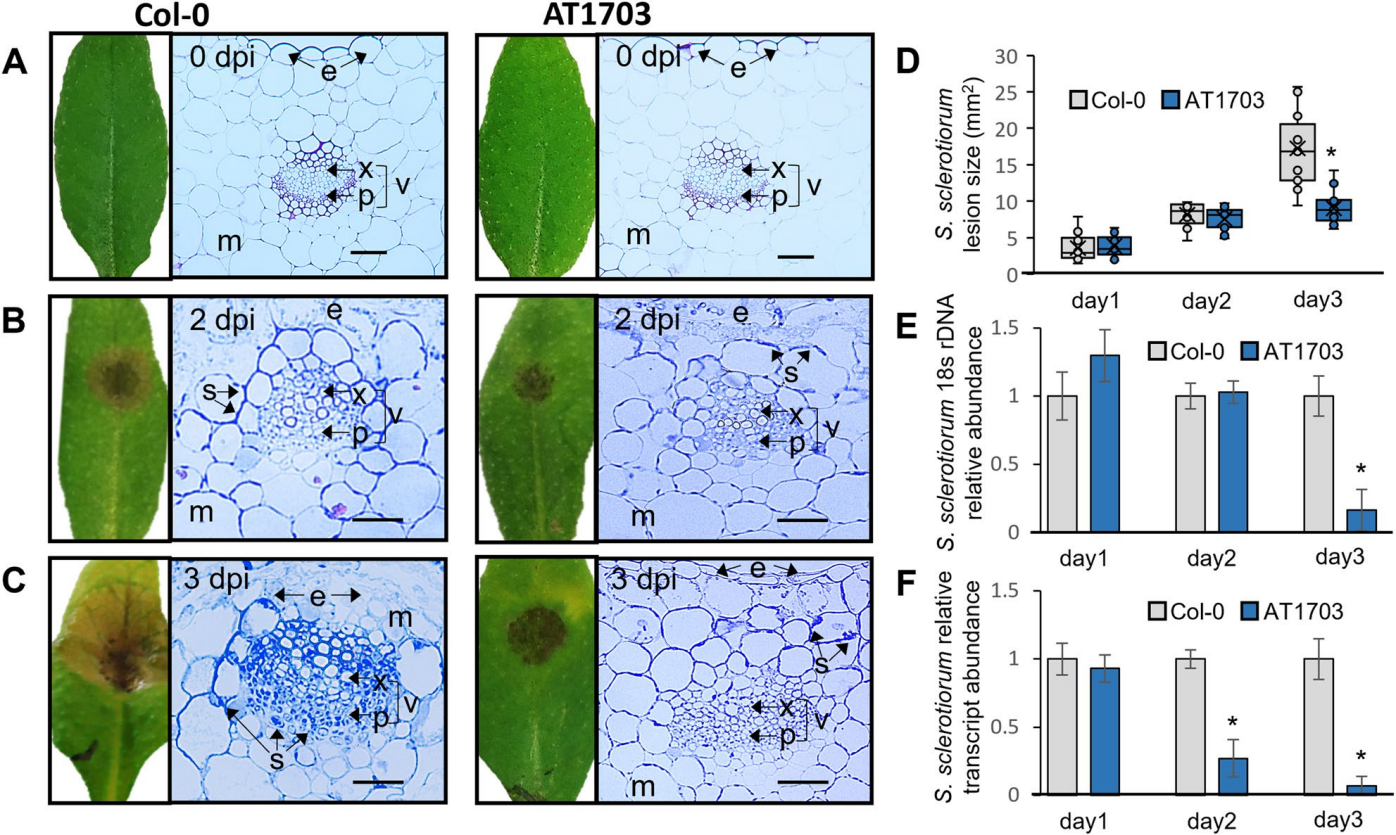
Whole-leaf and cross section of wild-type Col-0 and AT1703 *A. thaliana* uninfected control and infected with *S. sclerotiorum* at (**A**) 0, (**B**) 2, and (**C**) 3 days post inoculation. Five µM cross-sections generated using Leica RM2245 and stained with (**A**) toluidine blue and (**B**,**C**) lactophenol cotton blue. (**D**) lesion size (mm^2^), (**E**) fungal load and (**F**) target transcript reduction in Col-0 and T3 AT1703 leaves infected with *S. sclerotiorum*. *A. thaliana* inoculated with 1 × 10^6^ µL ascospore solution and measured at 2- and 3-days post inoculation. (**D**) Sample size of n = 15 leaves per line were measured using ImageJ software and normalized to wild-type Col-0 leaves for lesion measurements. Error bars represent standard error and significance was determined using a Student’s *t*-test (p < 0.05). * = significance. (**E**) Fungal load quantified using relative abundance of 18S *S. sclerotiorum* rDNA and calculated using ∆CT against wild-type Col-0. Each line consists of three biological replicates, each containing three infected *A. thaliana* leaves. (**F**) Target transcript abundance calculated using the ∆∆CT method with *Sac7* as a reference gene. Each line consists of three biological replicates, each containing three infected *A. thaliana* leaves for (**E**) and (**F**). Error bars represent standard error. Scale bars = 50 µm. e, epidermis; m, mesophyll; x, xylem; p, phloem; v, vasculature; s, *S. sclerotiorum.*

hpRNA complementary to the *ABHYDROLASE-3* gene coding sequence of *S. sclerotiorum* (SS1G_01703) was transformed into wild-type Col-0 *A. thaliana* under the control of the CaMV 35S promoter and propagated to the T3 generation. Three independent insertion lines were propagated to the T2 generation (Supplementary Fig. S1a), and our top performing line (AT1703.1, herein referred to as AT1703) was selected for RNA sequencing and further analysis at the T3 generation due to its significantly reduced lesion size and *ABHYDROLASE-3* transcript reduction compared to wild-type Col-0 (Supplementary Fig. S1b). Light microscopy of leaf cross sections using the chitin-targeting stain lactophenol blue showed no apparent differences in uninfected AT1703 and wild-type Col-0 leaves (Fig. 1a). However, at 2 dpi, wild-type Col-0 leaves show an increased abundance of *S. sclerotiorum* in both mesophyll and vascular tissues (Fig. 1b). At 3 dpi *S. sclerotiorum* shows further colonization of vascular tissue in wild-type Col-0 compared to AT1703 leaves, coupled with decreased levels of epidermal and mesophyll tissue degradation in AT1703 lines (Fig. 1c). At 1 dpi, no differences in lesion size or fungal abundance were observed (Fig. 1d,e), while at 2 dpi, *S. sclerotiorum ABHYDROLASE-3* transcript levels showed a 73% reduction in AT1703 with no significant differences in lesion size or fungal load compared to wild-type Col-0 (Fig. 1f). However, by 3 dpi AT1703 showed a 48% reduction in lesion size, an 84% reduction in fungal load, and a 93% reduction in *ABHYDROLASE-3* transcript accumulation compared to wild-type Col-0 plants (Fig. 1d,f). While this significant transcript reduction is present as early as 2 dpi, these data suggest a ~ 24-h lag phase where transcript abundance is reduced but insufficient to immediately slow infection (Fig. 1d,f).

### Global RNA-sequencing reveals enriched gene activity of the salicylic acid-mediated SAR pathway in AT1703

**Figure 2:**
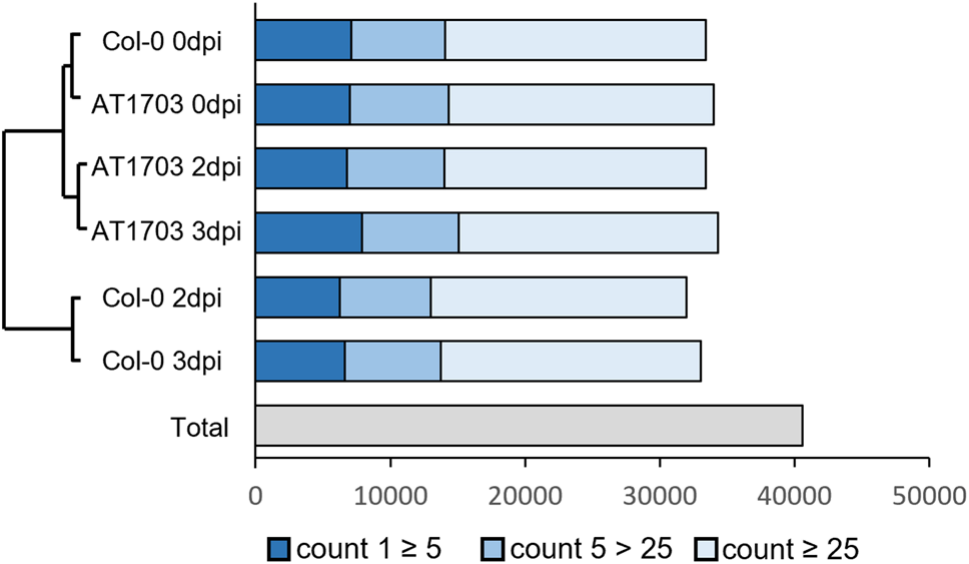
Global gene activity in the *A. thaliana-S. sclerotiorum* pathosystem. Hierarchical clustering and global gene activity of wild-type Col-0 and AT1703 at 0-, 2- and 3-days post inoculation*.* Number of transcripts detected across all treatments were identified through RNA sequencing. Transcripts with a count value > 1 are considered detected. Detected transcripts are subdivided into low (1 ≥ 5), moderate (5 > 25), or high (≥ 25) abundance levels.

To uncover the underlying gene expression patterns between AT1703 and wild-type Col-0 we performed global RNA sequencing on uninfected (0 dpi) leaves as well as directly at the site of infection at mid (2 dpi) and late (3 dpi) stages of *S. sclerotiorum* leaf infection. Hierarchical clustering of raw count expression values revealed distinct clusters forming between uninfected (0 dpi) and *S. sclerotiorum* infected tissues (Fig. 2). Genotypes also clustered in response to *S. sclerotiorum* infection compared to infection time points. Global gene activity showed no significant differences in the proportion of low, moderate or highly accumulated transcripts, with on average 20%, 21% and 58% of detected transcripts showing low, moderate and high-count levels respectively (Fig. 2). Further, total transcript detection between genotypes is similar, with an increase in 1813 and 1348 detected transcripts in AT1703 compared to Col-0 at 2 and 3 dpi respectively due to an increased abundance of *S. sclerotiorum* transcripts in wild-type Col-0.

**Figure 3:**
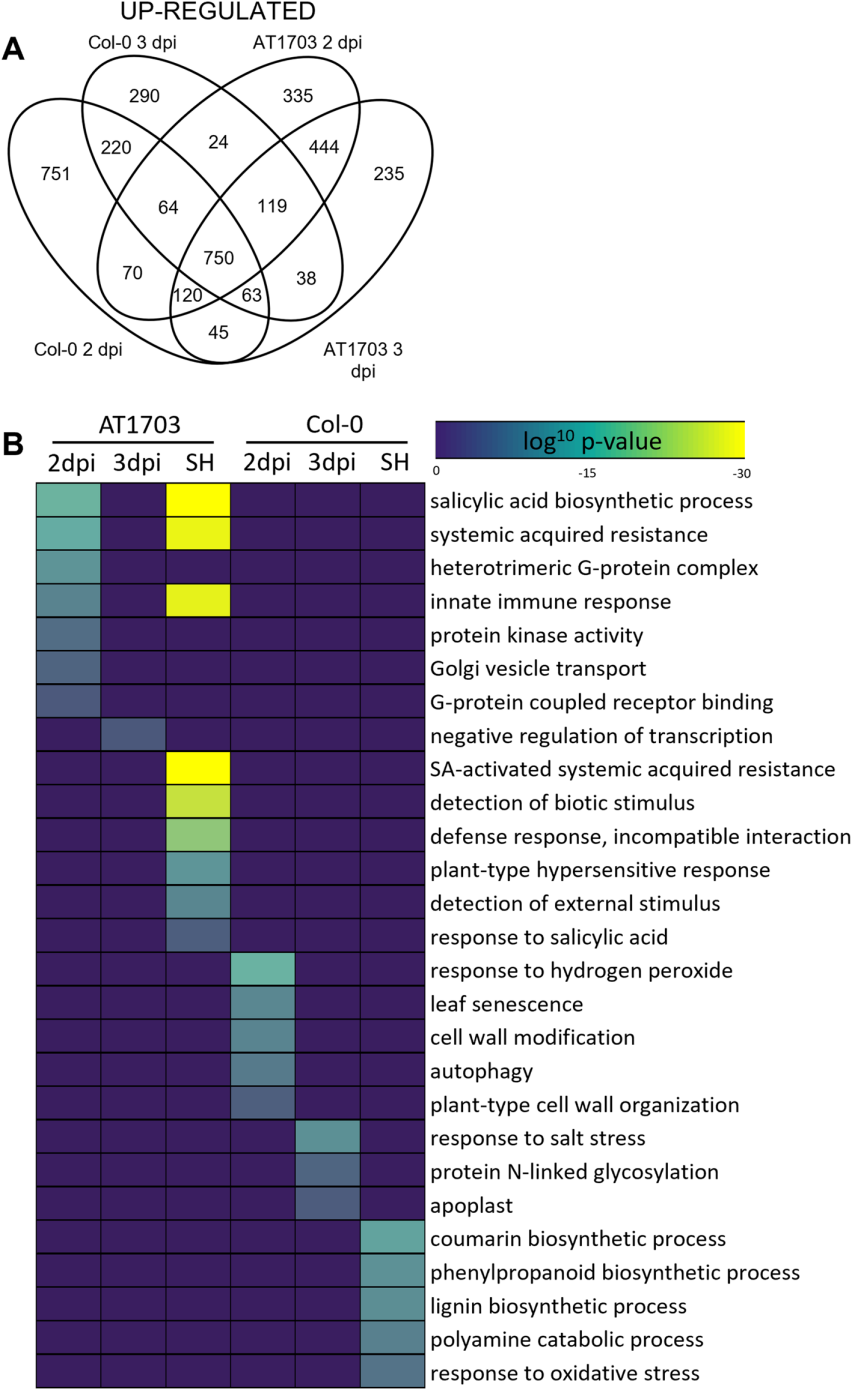
Identification of differentially expressed genes and enriched GO terms in response to *S. sclerotiorum* infection in wild-type Col-0 and AT1703. (**A**) Venn diagram representing up-regulated genes in infected wild-type Col-0 and AT1703 *A. thaliana*. (**B**) Heatmap of enriched GO terms associated with shared up-regulated gene patterns. GO terms considered statistically significant with a hypergeometric log10 p-value < 0.001. *SH* shared.

Differential expression analysis identified shared and specifically up-regulated gene sets in response to *S. sclerotiorum* in AT1703 and wild-type Col-0 (Fig. 3a). For example, 335 and 235 genes were specifically up-regulated at 2 and 3 dpi respectively, while 444 genes were up-regulated at both timepoints in AT1703. Further, 449 and 235 genes were down-regulated in AT1703 relative to Col-0 at 2 and 3 dpi respectively, and 351 genes were down-regulated at both timepoints (Supplementary Dataset S4). Next, to identify putative biological processes associated with these differentially expressed gene sets, we performed Gene ontology (GO) term enrichment (Fig. 3b). Data show the SA-mediated SAR pathway (p = 2.05 × 10^–27^) and SA biosynthesis (p = 1.16 × 10^–31^) as well as enrichment of the hypersensitive response (p = 3.07 × 10^–7^) in gene sets up-regulated at 2 and 3 dpi in AT1703. The enrichment of SA biosynthesis, SAR and the hypersensitive response indicate a strong role of the SAR defense pathway in successful defense response and incompatible interaction (p = 1.28 × 10^–13^) found in AT1703 lines. While SA biosynthesis and SAR demonstrate shared enrichment between AT1703 at 2 and 3 dpi, it is worth noting that statistically significant enrichment is also found specifically at 2 dpi, while no such enrichment is found for down-stream activated defense processes like the hypersensitive response and incompatible interaction (Fig. 3b). Further, 750 genes were up-regulated in response to *S. sclerotiorum* across both genotypes and timepoints (Fig. 3a). This shared gene set showed significant enrichment of PTI associated JA (p = 7.77 × 10^–34^) and ET biosynthesis (p = 1.32 × 10^–49^), induced systemic resistance (p = 6.63 × 10^–8^) and PAMP induction of immune response (p = 1.71 × 10^–4^) (Supplementary Dataset S1), suggesting that activity of these JA/ET-mediated defense pathways alone in wild-type Col-0 are insufficient to slow *S. sclerotiorum* infection without enrichment of SA-mediated SAR, providing additional evidence of the synergism of SA-mediated SAR and JA/ET-mediated ISR/PTI in mounting a successful defense against *S. sclerotiorum*. In contrast, enrichment of up-regulated genes specifically in wild-type Col-0 show association with programmed cell death and cell wall reinforcement, including leaf senescence (p = 1.97 × 10^–6^), autophagy (p = 8.88 × 10^–6^), cell wall modification (p = 2.61 × 10^–6^) and organization (p = 2.75 × 10^–4^), and lignin biosynthesis (p = 7.22 × 10^–7^).

**Figure 4:**
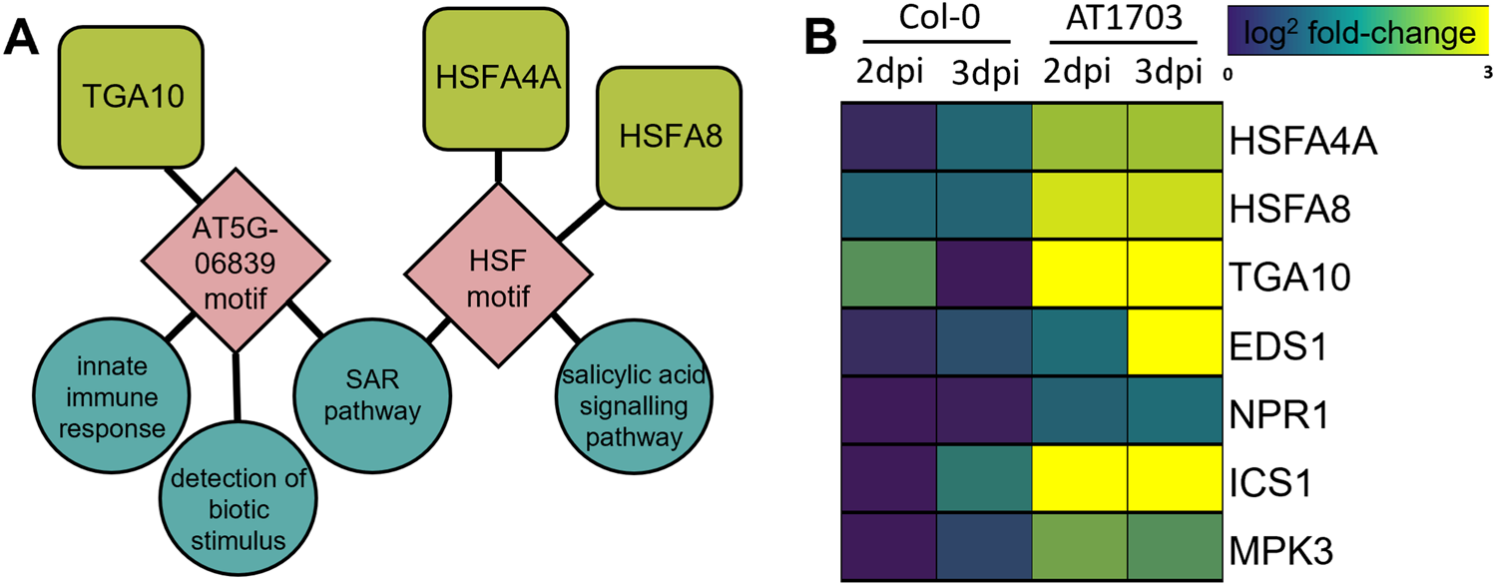
Differential expression and transcription factor network analysis of up-regulated shared AT1703 gene set in response to *S. sclerotiorum*. (**A**) Predictive transcription factor network analysis in shared AT1703 shared gene set in response to *S. sclerotiorum.* Yellow-green rounded squares represent significantly up-regulated transcription factors identified in AT1703 gene set, pink diamonds represent transcription factor DNA binding motifs, teal circles represent GO terms, and edges between DNA motifs and GO terms represent enriched motif sequences involved in the regulation of the GO term. (**B**) Log^2^ fold change of infected AT1703 and wild-type Col-0 at 2- and 3-days post inoculation relative to respective uninfected treatments*.*

To explore potential regulators of immunity in AT1703 expressed throughout infection, we performed a transcription factor network analysis using SeqEnrich in our AT1703 up-regulated gene set in response to *S. sclerotiorum.* Here, we identified the TGA transcription factor *TGA10*, as well as the HSFs transcription factors *HSFA4A* and *HSFA8,* which are both predicted to regulate SA-signalling and SAR activity (Fig. 4a). Further, we explored differential expression between AT1703 and wild-type Col-0 in response to *S. sclerotiorum* in genes that have been previously described to interact with these transcription factors. Here, we found up-regulation of the TGA interacting *ENHANCED DISEASE SUSCEPTIBILITY 1* (*EDS1*), *NONEXPRESSER OF PR GENES 1* (*NPR1*) and *ISOCHORISMATE SYNTHASE 1* (*ICS1*) in AT1703 *A. thaliana*, as well as up-regulation of the *HSFA4A* interacting kinase *MITOGEN-ACTIVATED PROTEIN KINASE 3* (*MPK3*) (Fig. 4b).

### *ABHYDROLASE-3* silencing influences expression of polyamine synthase genes in *S. sclerotiorum*

**Figure 5:**
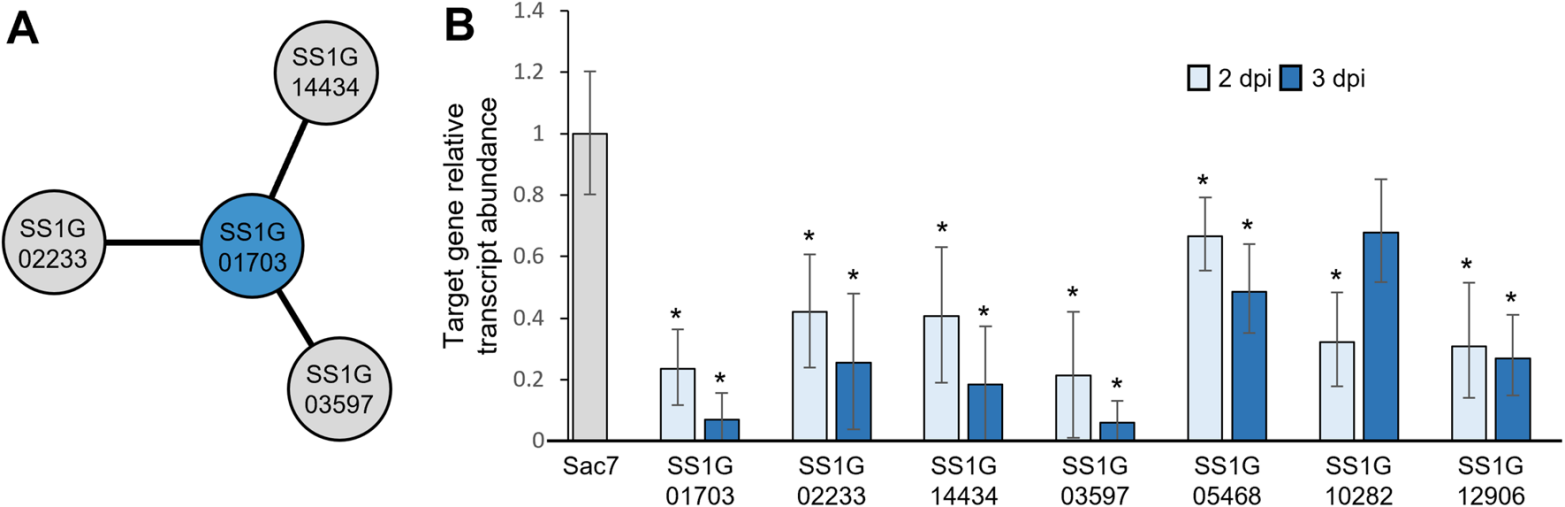
Transcript abundance of *ABHYDROLASE-3* (SS1G_01703) predicted interacting partners using RT-qPCR. (**A**) predictive interacting network of *ABHYDROLASE-3* generated through Stringdb based on gene fusion and co-expression analyses. (**B**) RT-qPCR target transcript abundance of predicted interacting partners in *S. sclerotiorum* challenging AT1703 *A. thaliana* at 2- and 3-days post inoculation relative to wild-type Col-0*.* Target transcript abundance calculated using the ∆∆CT method with *Sac7* as a reference gene. Each line consists of three biological replicates, each containing three infected *A. thaliana* leaves. Error bars represent standard error and significance was determined using a Student’s *t*-test (p < 0.05) with a Bonferroni correction. *Significance.

While AT1703 demonstrates significant *ABHYDROLASE-3* transcript reduction during *S. sclerotiorum* infection, we had yet to explore the influence of *ABHYDROLASE-3* silencing on gene expression within the fungus itself. Three predicted interacting partners of *ABHYDROLASE-3* (SS1G_02233, SS1G_14434, SS1G_03597) were identified in *S. sclerotiorum* using stringdb (https://string-db.org/) (Fig. 5a). While SS1G_14434 and SS1G_03597 have yet to be significantly characterized, SS1G_02233 encodes a 5′-methylthioadenosine phosphorylase (MTAP) that prevents accumulation of the polyamine biosynthesis by-product S-methyl-5′-thioadenosine (MTA), inhibiting polyamine biosynthesis and ultimately fungal growth^31,32^. Here, all three predicted interacting partners showed significant transcript reduction in *S. sclerotiorum* that was infecting AT1703 compared to wild-type Col-0 at 2 and 3 dpi (Fig. 5b). Further, reduction in these predictive partners was consistent with our target gene *ABHYDROLASE-3*, showing at least a 50% increase in reduction from 2 to 3 dpi. To further support the role of *ABHYDROLASE-3* in polyamine biosynthesis, we quantified expression of genes encoding the rate-limiting enzyme in polyamine biosynthesis *ORNITHINE DECARBOXYLASE* (*ODC*) as well as down-stream enzymes *SPERMINE SYNTHASE* and *SPERMIDINE SYNTHASE*. Transcript reduction was observed for all three genes in *S. sclerotiorum* challenging AT1703, suggesting a role of *ABHYDROLASE-3* in polyamine biosynthesis during *S. sclerotiorum* infection.

### AT1703 is not tolerant to the phytopathogen *Botrytis cinerea*

**Figure 6:**
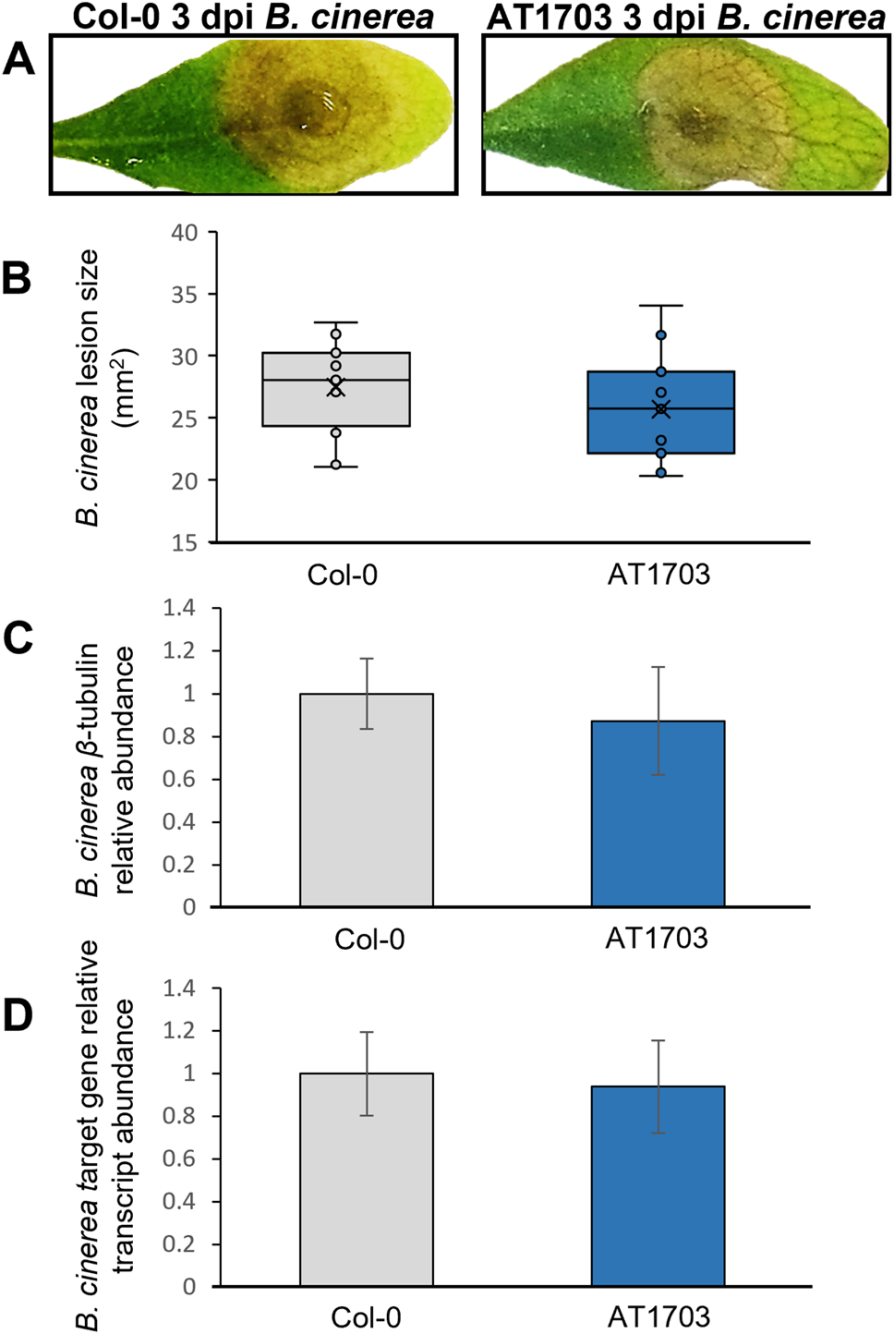
Lesion size (mm^2^), fungal load and target transcript knock down in wild-type Col-0 and T3 transgenic AT1703 *A. thaliana* leaves infected with *B. cinerea*. *A. thaliana* inoculated with 1 × 10^6^ µL ascospore solution and measured at 3 days post inoculation. (**B**) Sample size of n = 15 leaves per line were measured using ImageJ software and normalized to wild-type Col-0 leaves for lesion measurements. Error bars represent standard error and significance was determined using a Student’s *t*-test (p < 0.05). (**C**) Fungal load quantified using relative abundance of *B. cinerea β–tubulin* and calculated using ∆CT against wild-type Col-0. Each line consists of three biological replicates, each containing three infected *A. thaliana* leaves. (**D**) Target transcript knockdown calculated using the ∆∆CT method with *β–tubulin* as a reference gene. Each line consists of three biological replicates, each containing three infected *A. thaliana* leaves for (**C)** and (**D**). Error bars represent standard error.

*ABHYDROLASE-3* targeting hpRNA expressed by AT1703 was carefully designed to not share 21-nt siRNAs with transcripts of related species including the closely related necrotrophic pathogen *B. cinerea*. To study whether AT1703 was also tolerant to *B. cinerea*, we next inoculated both wild-type Col-0 and AT1703 with *B. cinerea* spores (Fig. 6a). Here, no significant differences in lesion size (Fig. 6b), fungal load (Fig. 6c) or target transcript abundance (Fig. 6d) were found using the *B. cinerea ABHYDROLASE-3* homolog BC1G_08022, suggesting no off-target effects in *B.cinerea* challenging AT1703.

## Discussion

RNAi technology has the demonstrated ability to control pathogenic fungi in host plants through constitutively expressed dsRNA molecules in transgenic plants via host-induced gene silencing (HIGS). The constitutive expression of fungal-targeting dsRNAs allows for a durable means of protection against pathogen attack throughout the host plant lifecycle^16,33,34^. Protection of the plant relies on successful uptake and processing of dsRNA and processed siRNAs which can be generated in the host prior to uptake by the challenging pathogen^3^. Cross-kingdom trafficking of both dsRNAs and processed siRNA has been demonstrated across the host–pathogen interface in necrotrophic pathogens including *S. sclerotiorum* and the closely related *B. cinerea,* making these species ideal candidates for control through RNAi^35,36^. Here, we engineered *A. thaliana* expressing hpRNA molecules complementary to the previously identified *ABHYDROLASE-3* in *S. sclerotiorum.*

Successful *S. sclerotiorum* infection involves the penetration and destruction of host epidermis and mesophyll tissue followed by colonization of the vasculature^28,37^. Once the vascular tissue has been colonized, infection is considered systemic, being able to cut off host nutrient supply while the fungus extends mycelia conveniently throughout host tissues. Susceptible genotypes have demonstrated preferential growth throughout the vascular tissue in *Brassica* species compared to respective tolerant lines^38^. While microscopy of *S. sclerotiorum* infected wild-type Col-0 and AT1703 demonstrates reduced *S. sclerotiorum* abundance in AT1703, we also find decreased levels of epidermal and mesophyll tissue degradation and reduced abundance of vascular tissue fungal colonization, thereby preventing systemic infection throughout the plant. Limiting systemic infection has significant implications on reducing yield losses from *S. sclerotiorum* infection. This was demonstrated by Wytinck et al*.*^16^, where transgenic *B. napus* lines expressing the same *ABHYDROLASE-3* hpRNA construct against *S. sclerotiorum* show reduced vascular tissue colonization coupled with an increase in seed mass compared to untransformed *B. napus* post whole-plant *S. sclerotiorum* infection. As infection typically initiates on mature leaves before progressing into stem tissue through the vascular system^39,40^, it is critical to reduce vascular colonization directly at the site of infection, which can ultimately slow *S. sclerotiorum* progression throughout the host.

Target gene silencing in the pathogen is essential to slow the infection through HIGS. AT1703 plants demonstrate significant *ABHYDROLASE-3* transcript reductions in *S. sclerotiorum* at 1 and 2 dpi, while reduced lesion size and fungal load are only identified at 3 dpi relative to wild-type Col-0 plants. Wytinck et al.^16^ showed similar results in transgenic *B. napus*, with significant reductions in *ABHYDROLASE-3* transcript abundance and fungal load observed at 1 and 2 dpi respectively. In contrast, prior HIGS studies targeting *S. sclerotiorum* essential genes (*TRX1*, *OA*) detected both target transcript reduction and reduced lesion size at 2 dpi in host plants^33,41^, suggesting this delay to be specific to HIGS plants targeting *ABHYDROLASE-3.* These findings may be attributed to down-stream interactions of *ABHYDROLASE-3* with other interacting partners. Reduced activity of *ABHYDROLASE-3* in addition to regulators of polyamine biosynthesis are likely responsible for or at least contribute to the inhibition of fungal growth. However, further characterization of *ABHYDROLASE-3* interacting partners and the polyamine biosynthesis pathway during *ABHYDROLASE-3* silencing are required to better understand this delay.

While the utility of HIGS in controlling *S. sclerotiorum* has been demonstrated in multiple host plants^16,33,34^, the underlying host response of these transgenic plants, as well as host–pathogen interactions in this system are less understood and dependent upon the specific gene being silenced. Here, global RNA sequencing and differential expression analyses provide insight into the response of AT1703 leaves to *S. sclerotiorum* directly at the site of infection. Moreover, the mode of pathogenicity of *S. sclerotiorum* has been recently debated, as emerging studies have suggested the presence of a brief biotrophic stage during early infection highlighted by the suppression of the SA-mediated SAR pathway^23,24^. SA biosynthesis in response to pathogen attack occurs through the isochorismate synthase (ICS) pathway, where *ICS1* is essential for SA biosynthesis and successful SAR^42^. Downstream of SA accumulation, *NPR1* activity is required for expression of pathogenesis-related (*PR*) genes involved in the SAR response through SA signal transduction^42,43^. AT1703 shows significant enrichment of the SA-mediated SAR pathway compared to wild-type Col-0, with enrichment of both *ICS1* and *NPR1* in response to *S. sclerotiorum* infection, suggesting an inability to suppress SAR while infecting AT1703 *A. thaliana*. Further, while *NPR1* does not contain a DNA binding domain, it acts as a transcriptional cofactor, enhancing TGA transcription factor binding affinity to *PR* genes upon translocation into the nucleus in response to SA^44^. While multiple TGA transcription factors have been shown to interact with *NPR1* in response to SA in *A. thaliana*^45^, *TGA10* interaction has yet to be explored. Here, *TGA10* was identified as the lone member of the TGA transcription factor family significantly enriched in AT1703 *A. thaliana* in response to *S. sclerotiorum* and was identified in our predictive transcription factor network to regulate SAR activity. Further, *TGA10* has been demonstrated to be necessary for activation of reactive oxygen species (ROS) response in PTI in response to the bacterial PAMP flg22, where *tga10* mutants were unable to activate flg22-response genes and showed reduced ROS production^46^, highlighting its importance in the activation of defense genes in response to pathogen attack. Our network analysis further identified two HSF transcription factors *(HSFA4A, HSFA8*) associated with the SA signalling pathway that are up-regulated in AT1703 in response to *S. sclerotiorum*. While HSFs have been shown to interact directly with *EDS1*, resulting in SA accumulation in response to pathogen attack^47,48^, similarly to *TGA10*, *HSFA4A* and *HSFA8* have specifically been implicated in the host ROS response as well as activation of the host antioxidant system^49,50^. Activation of these HSFs can act through phosphorylation by *MPK3* in response to stress, which also demonstrates significant up-regulation in AT1703 *A. thaliana* in response to *S. sclerotiorum*^49,51,52^.

While these data suggest SAR activity is an essential component of a successful defense response against *S. sclerotiorum* infection, SAR alone is insufficient to protect the host, as quantitative disease resistance is complex and requires the coordination of multiple defense pathways and processes^53^. For example, SA-mediated SAR and JA/ET regulated ISR pathways exhibit synergistic effects in defense against necrotrophic pathogen attack^29^. This was demonstrated using *npr1* knockout Arabidopsis mutants deficient in SA signalling, *coi1* mutants deficient in JA signalling, and *ein2* deficient in ET signalling, which all show hyper-susceptibility phenotypes due to the inactivity of SAR and ISR pathways^30^. Differential expression analysis not only identified enrichment of SA-mediated SAR in AT1703, but also JA/ET biosynthesis and the ISR pathway which were significantly enriched in both AT1703 and wild-type Col-0 lines relative to their respective uninfected leaves, suggesting both defense pathways are essential in mounting a successful defense response against *S. sclerotiorum*.

To better understand the different defense responses to infection by wild-type Col-0 and AT1703 plants, we further explored defense processes enriched specifically in wild-type Col-0 and not AT1703. *S. sclerotiorum* demonstrates necrotrophic growth, especially at later stages of infection. Thus, the bi-products of gene activity belonging to autophagy and leaf senescence found in wild-type Col-0 can serve as nutrients for the progressing pathogen to continue growth as it feeds on necrotic host tissue^54,55^. With increased necrotic host tissue available to *S. sclerotiorum* in wild-type Col-0, host plants would require mechanical defenses through cell wall reinforcement and lignification in response to cell wall degrading enzymes being consistently secreted during actively progressing infection^56^. Lignification in response to *S. sclerotiorum* has previously been described by Höch et al.^56^, where genes associated with lignin biosynthesis showed significantly increased expression levels in susceptible *B. napus* lines compared to moderately resistant lines, specifically during mid to late stages of infection. These findings agree with our data where we find enrichment of lignin biosynthesis and cell wall reinforcement in wild-type Col-0 lines compared to AT1703 throughout *S. sclerotiorum* infection.

While we have examined the influence *ABHYDROLASE-3* silencing has on the host response, there is also a need to explore differences within the pathogen and the effect that *ABHYDROLASE-3* silencing has at the molecular level. *ABHYDROLASE-3* is annotated as a tRNA synthetase but also predicted to be involved in aflatoxin biosynthesis. However, this gene has yet to be further characterized beyond its ability to slow *S. sclerotiorum* infection through SIGS and HIGS RNAi treatments^15,16^. Using the stringdb (https://string-db.org/) protein–protein interaction network database, we identified three predictive interacting partners of *ABHYDROLASE-3* (SS1G_14434, SS1G_03597 and SS1G_02233)*.* Expression levels of *ABHYDROLASE-3* and these predicted interacting partners in *S. sclerotiorum* were coordinately reduced in transgenic AT1703 compared to wild-type Col-0. While SS1G_14434 and SS1G_03597 have yet to be functionally characterized in *S. sclerotiorum*, SS1G_02233 encodes MTAP, which plays a critical role in cleaving and processing accumulated MTA, a by-product of polyamine biosynthesis^31,32^. Buildup of MTA through inactivity of MTAP ultimately results in the inhibition of polyamine biosynthesis^31,32^. This in turn can have significant impacts on fungal growth, as the three most widely distributed polyamines, putrescine, spermidine and spermine have been shown to be necessary for successful growth and cell differentiation^32,57^. The ornithine decarboxylase (SS1G_05468) is an essential enzyme of polyamine biosynthesis, converting ornithine into the polyamine putrescine^32,57^. Once ornithine is converted into putrescine, there are further down-stream synthase genes necessary for conversion into both spermine and spermidine. These enzymes include spermine synthase (SS1G_12906) to convert putrescine into spermine, and spermidine synthase (SS1G_10282) to convert spermine into spermidine. Our RT-qPCR analysis of these essential polyamine synthesis genes shows transcript reduction during *S. sclerotiorum* infection of AT1703 plants compared to their wild type susceptible counterparts, suggesting a potential interaction between *ABHYDROLASE-3* and the identified predictive partners of the polyamine biosynthesis pathway. Further, as putrescine can also be produced through spermine and spermidine catabolism, expression of rate limiting enzymes SSAT and PAO can also be explored to improve our understanding of *ABHYDROLASE-3* influence on polyamine metabolism^32^. While the potential direct or indirect interaction of *ABHYDROLASE-3* and these predictive interacting partners is still unclear, these data provide some preliminary insights of the role *ABHYDROLASE-3* plays in *S. sclerotiorum* pathogenicity and further identifies a group of promising RNAi candidate genes through the polyamine synthesis pathway.

One benefit of RNAi and HIGS technology in crop protection is the ability to design pathogen specific molecules that are ineffective against potentially beneficial species in the ecosystem^4,58^. This requires the careful design of target sequences within genes, as dsRNA molecules containing as few as three overlapping 21-mer nucleic acid sequences have demonstrated the ability to significantly reduce off-target transcripts^59^. *B. cinerea* serves as an ideal candidate to study these potential off-target effects in controlling *S. sclerotiorum* given both pathogens share similar hosts and disease cycles in addition to having a high level of sequence homology^60,61^. McLoughlin et al.^15^ showed that dsRNA targeting the *ABHYDROLASE-3 B. cinerea* homolog BC1G_08022 was able to reduce *B. cinerea* lesion size, fungal load and BC1G_08022 transcript abundance when applied to *B. napus* leaves prior to *B. cinerea* inoculation. While BC1G_08022 is capable of slowing *B. cinerea* infection when targeted, our dsRNA region targeting *S. sclerotiorum ABHYDROLASE-3* shows no 21-mer overlapping regions with off-target species (Supplementary Dataset S2) and AT1703 *A. thaliana* challenged with the closely related necrotrophic fungal pathogen *B. cinerea* shows no significant differences in lesion size, fungal load or BC1G_08022 transcript reduction.

Taken together, these data demonstrate the efficacy of HIGS as an alternative control measure in slowing *S. sclerotiorum* infection through targeted silencing of *S. sclerotiorum ABHYDROLASE-3*. Reduction of *ABHYDROLASE-3* in *S. sclerotiorum* provides *A. thaliana* the ability to increase innate defense responses through SA-mediated SAR at the global mRNA level. The identification of co-silenced *ABHYDROLASE-3* interacting partners further uncovers a promising group of candidates for RNAi control against fungal pathogens. Finally, with ongoing concerns of potential off-target effects in RNA-based applications, we demonstrate the specificity of HIGS targeting *S. sclerotiorum* in AT1703 to a closely related fungus. Taken together, we provide evidence that HIGS applications can be used as an effective and sustainable strategy in crop improvement against necrotrophic pathogens.

## Methods

### Generation of transgenic *Arabidopsis*

DNA constructs consisted of a CaMV-35S promoter driving expression of a sense and antisense SS1G_01703 gene fragment separated by an intron region to form hpRNA post transcription. Cloning of this construct into *Agrobacterium tumefaciens* followed the Gateway cloning protocol (Invitrogen, Carlsbad, CA, US) using primers found in Supplementary Dataset S3 and following the methods of^16^. Target gene sequences were amplified using Phusion Taq (Thermo Scientific, Waltham, MA, US) under the following conditions: 98 °C for 30 s; 35 cycles of: 98 °C for 10 s, 57 °C for 30 s, and 72 °C for 30 s; and a final extension of 72 °C for 5 min. Amplicons were gel purified (New England Biolabs, Ipswich, MA, US) and digested using FastDigest *KpnI* and *XhoI* (Thermo Scientific, Waltham, MA, US) according to the manufacturer’s protocols. Initially, gene fragments were ligated into the pENTR4 vector before insertion into the pHellsgate8 destination vector. To confirm insert identity, entry vectors were sequenced at the Centre for Applied Genomics in Toronto, Ontario. Inserts were then recombined into the destination vector using a Gateway LR clonase reaction (ThermoFisher Scientific) following the manufacturer’s instructions, with the modification of adding 4:1 entry vector to destination vector ratio according to Wytinck et al.^16^. A cold shock treatment was used to transform Agrobacterium. Successful transformants were selected using colony PCR with *XhoI* and *XbaI* separate restriction enzyme digestions. Cultures of *Agrobacterium* were grown to OD 1.6–2.0, pelleted using centrifugation, and re-suspended in MS media and 0.001% Silwet L-77. To transform *A. thaliana*, seeds were initially sterilized using alternating washes of 70% and 95% ethanol then placed on Murashige and Skoog (MS) media (Sigma Aldrich). Seeds were vernalized for three days at 4 °C prior to being grown in a constant light incubator. Upon formation of first leaves *A. thaliana* seedlings were transplanted into Sunshine Mix #1 and grown to maturity at 22 °C. Mature flowering *A. thaliana* plants were dipped into the *Agrobacterium* culture and kept in high humidity conditions for two days. Floral dips were repeated five times before seeds were dried and harvested. Transformants were selected using MS and 150 μg/mL kanamycin media^62^. PCR was used to confirm the presence of the *S. sclerotiorum* gene using the same primers used for cloning within the plant (Supplementary Dataset S3). Three confirmed independently transformed *A. thaliana* lines were grown to the T2 generation and tested for their ability to silence *ABHYDROLASE-3* transcripts and slow *S. sclerotiorum* infection. The top performing line (AT1703.1) was subsequently selected for further RNA sequencing analysis.

### *Arabidopsis thaliana* infection assays

*Sclerotinia sclerotiorum* ascospores were collected at the Morden Research and Development Centre, Agriculture and Agri-Food Canada, Morden, MB, Canada and stored at 4 °C in desiccant in the dark according to Ref.^15^. *S. sclerotiorum* ascospore inoculum was made by suspending a 1 × 10^6^ spore/mL concentration of spores in a potato dextrose broth (PDB) and peptone solution (24 g PDB, 10 g peptone, 1 L water). Using a pipette, 10 µL of the solution was transferred onto mature transgenic and wild-type Col-0 *A. thaliana* leaves at a n = 15 leaves per treatment. Plants were stored in growth chambers at room temperature (21.0 °C), with plant trays being sealed with lids to maintain high levels of humidity for three days, allowing for infection progression in planta. At 2- and 3-days post inoculation (dpi), lesion area was quantified using ImageJ software and leaves were collected for RNA sequencing, fungal load, and transcript abundance experiments. Fifteen infection sites were quantified per treatment while three leaves were collected per bio-replicate with three biological replicates collected per treatment. *Botrytis cinerea* infections were performed using ascospores from the Saint-Jean sur Richelieu Research and Development Centre, Agriculture and Agri-Food Canada, QC, Canada following the same protocol for *S. sclerotiorum* ascospore inoculations.

### DNA extraction and plant genotyping

Two-week-old *A. thaliana* leaves were collected and ground using a GenoGrinder 2000 (Spex CertiPrep, Metuchen, New Jersey, USA). DNA was extracted according to Wytinck et al.^16^ with DNA extraction buffer [0.25 M NaCl, 1 M 2-amino-2-(hydroxymethyl)-1,3-propanediol (TRIS)-HCl pH 7.5, 25 mM EDTA pH 8, 0.5% SDS]. DNA was precipitated with isopropanol and subsequently washed with 75% ethanol before being dissolved in water. PCR was performed using GoTaq^®^ Green Master Mix (Promega, Madison WI, USA) according to manufacturer’s instructions using *ABHYDROLASE-3* specific primers (Supplementary Dataset S3). Thermocycler conditions were 94 °C for 2 min. followed by 35 cycles of: 94 °C for 1 min, 56 °C for 30 s, 72 °C for 35 s. and a final extension at 72 °C for 5 min. PCR products were then loaded on a 1% agarose gel containing ethidium bromide with 2 μL of sample loaded per well against FastRuler Middle Range DNA Ladder (Thermo Fisher) as a reference. Gels were visualized under UV light using the Axygen^®^ Gel Documentation System (Axygen, Corning, NY, US).

### Lactophenol cotton blue stain microscopy

*Arabidopsis thaliana* leaf tissue was embedded in historesin (Leica, Wetzlar, GER) and vacuum infiltrated in 2.5% glutaraldehyde and 1.6% paraformaldehyde fixative solution. Methylcellosolve (Sigma-Aldrich, St. Louis, MO, USA) was used to remove pigment followed by three transfers of 100% ethanol, one every 24 h to dehydrate tissue. Tissue was then infiltrated with historesin during a 3-day period of increasing concentrations of resin. Day 1: 1:3 historesin:100% alcohol, day 2: 2:3 historesin:100% alcohol, and day 3: 100% historesin. Tissue was then embedded in blocks with an embedding solution of activated historesin, hardener and polyethylene glycol according to Wytinck et al.^16^. Sections were cut at 5 μM with a Leica RM2245 microtome and *S. sclerotiorum* infected tissues stained with lactophenol cotton blue for 20 min, while uninfected 0 days post inoculated tissues were stained with 0.1% toluidine blue O for 15 min. Uninfected and infected wild-type Col-0 and AT1703 *A. thaliana* leaves were imaged using a Leica DFC450C camera.

### RT-qPCR analysis

*Sclerotinia sclerotiorum* and *B. cinerea* RT-qPCR primers were designed using Primer3 (http://bioinfo.ut.ee/primer3/) and subsequently validated using the Primer BLAST tool (www.ncbi.nlm.nih.gov/tools/primer-blast) with a product range of 70–90 bp, GC% range of 40–60% and a primer size range of 18–23 bp. Transcript abundance was measured on the Bio-Rad CFX96 Connect Real-Time system using SsoFast EvaGreen Supermix (Bio-rad Laboratories, Hercules, CA, US) in 10 µL reactions according to manufacturer’s protocol using the following conditions: 95 °C for 30 s, and 45 cycles of: 95 °C for 2 s and 60 °C for 5 s. Melt curves were performed at a range of 65–95 °C with 0.5 °C increments to assess nonspecific amplification and primer dimers. Relative transcript accumulation was calculated using the ΔΔCt method^63^, relative to *Sac7* (SS1G_12350) for *S. sclerotiorum* and *tubA* (BC1G_00122) for *B. cinerea* using three biological replicates per treatment and three technical replicates per biological replicate*.* The same ΔΔCt method was used to quantify expression levels of RNAi machinery in *S. sclerotiorum* and have been used and validated in Refs.^15,16^. All RT-qPCR primers used in this study can be found in Supplementary Dataset S3.

### mRNA library preparation and RNA sequencing

RNA was isolated using the Ambion^®^ RNaqueous^®^ micro kit (ThermoFisher Scientific) according to manufacturer’s instructions. RNA quality was analyzed using the Agilent 6000 Pico LabChip^®^ and Agilent 2100 Bioanalyzer software (Agilent Technologies, USA). RNA quantity was determined using the Quant-iT™ RiboGreen^®^ kit (ThermoFisher Scientific) according to manufacturer’s instructions. NEBNext Oligo d(T)_25_ beads (New England BioLabs, Ipswich, MA, USA) were used for mRNA purification. cDNA library synthesis was performed according to Ziegler et al.^64^ with three biological replicates per treatment each containing three *A. thaliana* leaves. Subsequently, 100 bp paired-end RNA sequencing was performed on the Illumina HiSeq4000 platform (Génome Québec Innovation Centre, McGill University, Montreal, Canada).

### RNA sequencing analysis

Raw and processed sequence reads were deposited at the Gene Expression Omnibus under the accession GSE217513. Read quality control and adapter sequence removal was performed using Trimmomatic 0.36^65^ (HEADCROP:9 LEADING:30 TRAILING:30 SLIDINGWINDOW:4:30 MINLEN:50). Surviving reads were aligned to the TAIR10 *A. thaliana* genome using HISAT2^66^ alignment software. Raw count data was generated using featureCounts^67^ and inputted into pvclust (https://cran.r-project.org/web/packages/pvclust/index.html) for hierarchical clustering analysis with a detection cutoff ≥ 1. Detected transcripts were subsequently sorted into low (1 ≥ 5), moderate (5 > 25), or high (≥ 25) abundance levels. Further, raw count data was processed in DESeq2 for differential expression analysis. Here, differentially expressed genes (DEGs) were identified between pairwise comparisons at a p-value cutoff of p < 0.0001. GO enrichment of identified DEGs was performed using SeqEnrich^68^ with terms being considered significantly enriched at p ≤ 0.001 according to software specifications. GO heatmap visualization was performed using the conditional formatting function in Excel. GO summary tables can be found in Supplementary Dataset S1. Predictive transcription factor networks were generated using SeqEnrich (Supplementary Dataset S5) with DEG gene lists being used as input. Networks were subsequently visualized using Cytoscape 3.9.1. software (https://cytoscape.org/).

## Supplementary Information

Supplementary Information 1.

Supplementary Information 2.

Supplementary Information 3.

Supplementary Information 4.

Supplementary Information 5.

Supplementary Information 6.

Supplementary Figure S1.

Supplementary Legends.

## Data Availability

All RNA sequencing data are available at the Gene Expression Omnibus (GEO) data repository (GSE217513).

## References

[1] Gutbrod MJ, Martienssen RA (2020). Conserved chromosomal functions of RNA interference. Nat. Rev. Genet..

[2] Agrawal N (2003). RNA interference: Biology, mechanism, and applications. Microbiol. Mol. Biol. Rev..

[3] Koch A, Wassenegger M (2021). Host-induced gene silencing—Mechanisms and applications. New Phytol..

[4] Zand Karimi H, Innes RW (2022). Molecular mechanisms underlying host-induced gene silencing. Plant Cell.

[5] Christiaens, O. *et al*. Literature review of baseline information on RNAi to support the environmental risk assessment of RNAi-based GM plants. *EFSA Support. Publ.* **15**(5) (2018).

[6] Patel RM, van Kan JA, Bailey AM, Foster GD (2008). RNA-mediated gene silencing of superoxide dismutase (bcsod1) in *Botrytis cinerea*. Phytopathology.

[7] McDonald T, Brown D, Keller NP, Hammond TM (2005). RNA silencing of mycotoxin production in *Aspergillus* and *Fusarium* species. Mol. Plant Microbe Interact..

[8] Song Y, Thomma BPHJ (2018). Host-induced gene silencing compromises *Verticillium wilt* in tomato and *Arabidopsis*. Mol. Plant Pathol..

[9] Laurie JD, Linning R, Bakkeren G (2008). Hallmarks of RNA silencing are found in the smut fungus *Ustilago hordei* but not in its close relative *Ustilago maydis*. Curr. Genet..

[10] Lawrence GJ, Dodds PN, Ellis JG (2010). Transformation of the flax rust fungus, *Melampsora lini*: Selection via silencing of an avirulence gene. Plant J..

[11] Latijnhouwers M, Govers F (2003). A *Phytophthora infestans* G-protein beta subunit is involved in sporangium formation. Eukaryot. Cell.

[12] Gaulin E, Jauneau A, Villalba F, Rickauer M, Esquerre-Tugaye MT, Bottin A (2002). The CBEL glycoprotein of *Phytophthora parasitica var-nicotianae* is involved in cell wall deposition and adhesion to cellulosic substrates. J. Cell Sci..

[13] Rajam MV, Chauhan S, Sarmah BK, Borah BK (2021). Host-induced gene silencing (HIGS): An emerging strategy for the control of fungal plant diseases. Genome Engineering for Crop Improvement. Concepts and Strategies in Plant Sciences.

[14] Nunes CC, Dean RA (2012). Host-induced gene silencing: A tool for understanding fungal host interaction and for developing novel disease control strategies. Mol. Plant Pathol..

[15] McLoughlin AG (2018). Identification and application of exogenous dsRNA confers plant protection against *Sclerotinia sclerotiorum* and *Botrytis cinerea*. Sci. Rep..

[16] Wytinck, N. *et al*. Host induced gene silencing of the *Sclerotinia sclerotiorum ABHYDROLASE-3* gene reduces disease severity in *Brassica napus*. *PLoS One*. **17**(8), (2022). 10.1371/journal.pone.0261102 PMC9417021 36018839

[17] Shahoveisi F, Riahi Manesh M, del Río Mendoza LE (2022). Modeling risk of *Sclerotinia sclerotiorum*-induced disease development on canola and dry bean using machine learning algorithms. Sci. Rep..

[18] Jamaux I, Gélie B, Lamarque C (1995). Early stages of infection of rapeseed petals and leaves by *Sclerotinia sclerotiorum* revealed by scanning electron microscopy. Plant. Pathol..

[19] Ghozlan MH, El-Argawy E, Tokgoz S, Lakshman DK, Mitra A (2020). Plant defense against necrotrophic pathogens. Am. J. Plant Sci..

[20] Kamle, M., Borah, R., Bora, H., Jaiswal, A. K., Singh, R. K. & Kumar P. Systemic acquired resistance (SAR) and induced systemic resistance (ISR): Role and mechanism of action against phytopathogens. In *Fungal Biotechnology and Bioengineering.* 457–470 (Springer, 2020).

[21] Shen Q, Liu L, Wang L, Wang Q (2018). Indole primes plant defense against necrotrophic fungal pathogen infection. PLoS ONE.

[22] Vlot AC (2021). Systemic propagation of immunity in plants. New Phytol..

[23] Allan J (2019). The host generalist phytopathogenic fungus *Sclerotinia sclerotiorum* differentially expresses multiple metabolic enzymes on two different plant hosts. Sci. Rep..

[24] Seifbarghi S (2017). Changes in the *Sclerotinia sclerotiorum* transcriptome during infection of *Brassica napus*. BMC Genom..

[25] Bolton MD, Thomma BP, Nelson BD (2006). *Sclerotinia sclerotiorum* (Lib.) de Bary: Biology and molecular traits of a cosmopolitan pathogen. Mol. Plant Pathol..

[26] Fagundes-Nacarath IRF, Debona D, Rodrigues FA (2018). Oxalic acid-mediated biochemical and physiological changes in the common bean-*Sclerotinia sclerotiorum* interaction. Plant Physiol. Biochem..

[27] Lyu X (2016). A small secreted virulence-related protein is essential for the necrotrophic interactions of *Sclerotinia sclerotiorum* with its host plants. PLoS Pathog..

[28] Walker PL (2022). Tissue-specific mRNA profiling of the *Brassica napus–Sclerotinia sclerotiorum* interaction uncovers novel regulators of plant immunity. J. Exp. Bot..

[29] Ngou BPM, Ahn H-K, Ding P, Jones JD (2021). Mutual potentiation of plant immunity by cell-surface and intracellular receptors. Nature.

[30] Guo X, Stotz HU (2007). Defense against *Sclerotinia sclerotiorum* in *Arabidopsis* is dependent on jasmonic acid, salicylic acid, and ethylene signaling. Mol. Plant Microbe Interact..

[31] Waduwara-Jayabahu I (2012). Recycling of methylthioadenosine is essential for normal vascular development and reproduction in *Arabidopsis*. Plant Physiol..

[32] Valdés-Santiago L, Ruiz-Herrera J (2014). Stress and polyamine metabolism in fungi. Front. Chem..

[33] McCaghey M (2021). Host-induced gene silencing of a *Sclerotinia sclerotiorum oxaloacetate acetylhydrolase* using bean pod mottle virus as a vehicle reduces disease on soybean. Front. Plant Sci..

[34] Wu J (2022). Host-induced gene silencing of multiple pathogenic factors of *Sclerotinia sclerotiorum* confers resistance to *Sclerotinia* rot in *Brassica napus*. Crop J..

[35] Wang M, Weiberg A, Lin FM, Thomma BP, Huang HD, Jin H (2016). Bidirectional cross-kingdom RNAi and fungal uptake of external RNAs confer plant protection. Nat. Plants..

[36] Wytinck N, Manchur CL, Li VH, Whyard S, Belmonte MF (2020). dsRNA uptake in plant pests and pathogens: Insights into RNAi-based insect and fungal control technology. Plants..

[37] Girard IJ (2017). RNA sequencing of *Brassica napus* reveals cellular redox control of *Sclerotinia* infection. J. Exp. Bot..

[38] Uloth MB, Clode PL, You MP, Barbetti MJ (2016). Attack modes and defence reactions in pathosystems involving *Sclerotinia sclerotiorum*, *Brassica carinata*, *B. juncea* and *B. napus*. Ann. Bot..

[39] Young CS, Werner CP (2011). Infection routes for *Sclerotinia sclerotiorum* in apetalous and fully petalled winter oilseed rape. Plant. Pathol..

[40] Davidson AL, Blahut-Beatty L, Itaya A, Zhang Y, Zheng SD (2016). Histopathology of *Sclerotinia sclerotiorum* infection and oxalic acid function in susceptible and resistant soybean. Plant. Pathol..

[41] Rana K (2021). *Sclerotinia sclerotiorum* Thioredoxin1 (SsTrx1) is required for pathogenicity and oxidative stress tolerance. Mol. Plant Pathol..

[42] Gao QM, Zhu S, Kachroo P, Kachroo A (2015). Signal regulators of systemic acquired resistance. Front. Plant Sci..

[43] Kinkema M, Fan W, Dong X (2000). Nuclear localization of NPR1 is required for activation of PR gene expression. Plant Cell.

[44] Kumar P (2018). Pivotal role of bZIPs in amylose biosynthesis by genome survey and transcriptome analysis in wheat (*Triticum aestivum* L.) mutants. Sci. Rep..

[45] Backer R, Naidoo S, van den Berg N (2019). The NONEXPRESSOR OF PATHOGENESIS-RELATED GENES 1 (NPR1) and related family: Mechanistic insights in plant disease resistance. Front. Plant Sci..

[46] Noshi M, Mori D, Tanabe N, Maruta T, Shigeoka S (2016). *Arabidopsis* clade IV TGA transcription factors, TGA10 and TGA9, are involved in ROS-mediated responses to bacterial PAMP flg22. Plant Sci..

[47] Wei Y, Liu G, Chang Y, He C, Shi H (2018). Heat shock transcription factor 3 regulates plant immune response through modulation of salicylic acid accumulation and signalling in cassava. Mol. Plant Pathol..

[48] Cui H, Gobbato E, Kracher B, Qiu J, Bautor J, Parker JE (2017). A core function of EDS1 with PAD4 is to protect the salicylic acid defense sector in *Arabidopsis* immunity. New Phytol..

[49] Khan A, Khan V, Pandey K, Sopory SK, Sanan-Mishra N (2022). Thermo-priming mediated cellular networks for abiotic stress management in plants. Front. Plant Sci..

[50] Qu AL, Ding YF, Jiang Q, Zhu C (2013). Molecular mechanisms of the plant heat stress response. Biochem. Biophys. Res. Commun..

[51] Andrási N (2019). The mitogen-activated protein kinase 4-phosphorylated heat shock factor A4A regulates responses to combined salt and heat stresses. J. Exp. Bot..

[52] Pérez-Salamó I (2014). The heat shock factor A4A confers salt tolerance and is regulated by oxidative stress and the mitogen-activated protein kinases MPK3 and MPK6. Plant Physiol..

[53] Wang Z (2019). Recent advances in mechanisms of plant defense to *Sclerotinia sclerotiorum*. Front. Plant Sci..

[54] Shao D, Smith DL, Kabbage M, Roth MG (2021). Effectors of plant necrotrophic fungi. Front. Plant Sci..

[55] Rajarammohan S (2021). Redefining plant-necrotroph interactions: The thin line between hemibiotrophs and necrotrophs. Front. Microbiol..

[56] Höch K, Koopmann B, von Tiedemann A (2021). Lignin composition and timing of cell wall lignification are involved in *Brassica napus* resistance to stem rot caused by *Sclerotinia sclerotiorum*. Phytopathology.

[57] Valdés-Santiago L, Cervantes-Chávez JA, León-Ramírez CG, Ruiz-Herrera J (2012). Polyamine metabolism in fungi with emphasis on phytopathogenic species. J. Amino Acids.

[58] Mcloughlin AG, Walker PL, Wytinck N, Sullivan DS, Whyard S, Belmonte MF (2018). Developing new RNA interference technologies to control fungal pathogens. Can. J. Plant Path..

[59] Good RT (2016). OfftargetFinder: A web tool for species-specific RNAi design. Bioinformatics.

[60] Mbengue M (2016). Emerging trends in molecular interactions between plants and the broad host range fungal pathogens *Botrytis cinerea* and *Sclerotinia sclerotiorum*. Front. Plant Sci..

[61] Reich J, Chatterton S, Johnson D (2017). Temporal dynamics of *Botrytis cinerea* and *Sclerotinia sclerotiorum* in seed alfalfa fields of southern Alberta, Canada. Plant Dis..

[62] Logemann E, Birkenbihl RP, Ülker B, Somssich IE (2006). An improved method for preparing *Agrobacterium* cells that simplifies the *Arabidopsis* transformation protocol. Plant Methods.

[63] Livak KJ, Schmittgen TD (2001). Analysis of relative gene expression data using real-time quantitative PCR and the 2(-Delta Delta C(T)) Method. Methods.

[64] Ziegler DJ, Khan D, Kalichuk JL, Becker MG, Belmonte MF (2019). Transcriptome landscape of the early *Brassica napus* seed. J. Integr. Plant Biol..

[65] Bolger AM, Lohse M, Usadel B (2014). Trimmomatic: A flexible trimmer for illumina sequence data. Bioinformatics.

[66] Kim D, Langmead B, Salzberg SL (2015). HISAT: A fast spliced aligner with low memory requirements. Nat. Methods.

[67] Liao Y, Smyth GK, Shi W (2014). featureCounts: An efficient general purpose program for assigning sequence reads to genomic features. Bioinformatics.

[68] Becker MG, Walker PL, Pulgar-Vidal NC, Belmonte MF (2017). SeqEnrich: A tool to predict transcription factor networks from co-expressed *Arabidopsis* and *Brassica napus* gene sets. PLoS ONE.

