# Supplementary material for: Control of white mold (Sclerotinia sclerotiorum) through plant-mediated RNA interference
Source: Sci Rep. 2023 Apr 20;13:6477. doi: 10.1038/s41598-023-33335-4 (PMC10119085; doi:10.1038/s41598-023-33335-4)
Supplement: Supplementary file 7 — Supplementary Figure S1. [file 41598_2023_33335_MOESM7_ESM.pdf]

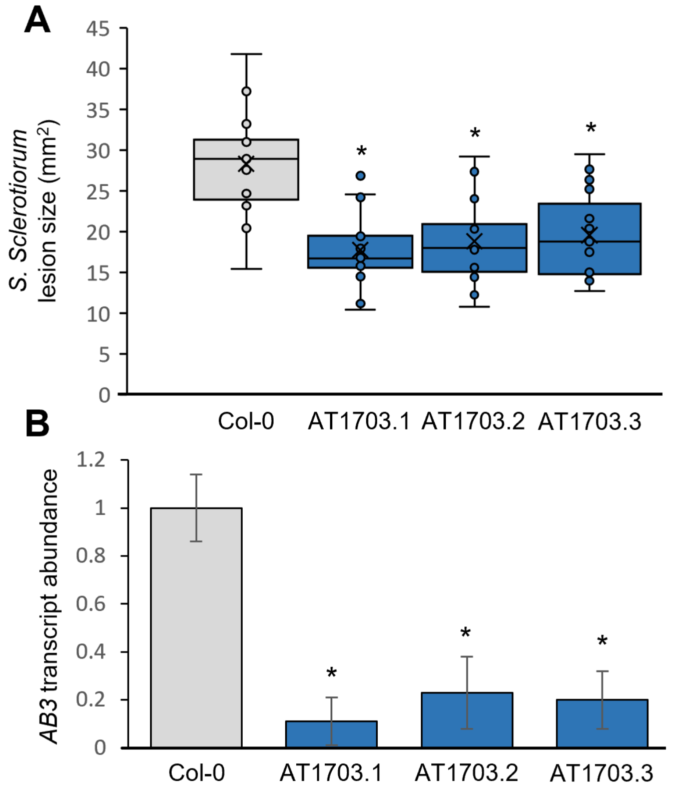

Figure S1. *S. sclerotiorum* lesion size and *AB3* transcript abundance in individual T2 generation AT1703 lines. A) Sample size of n = 15 leaves per line were measured using ImageJ software and normalized to wild-type Col-0 leaves for lesion measurements. B) Target transcript abundance calculated using the ΔΔCT method with Sac7 as a reference gene. Each line consists of 3 biological replicates, each containing three infected *A. thaliana* leaves. Error bars represent standard error and significance was determined using a student's t-test ($p<0.05$). * = significance.
