# Supplementary material for: Control of white mold (Sclerotinia sclerotiorum) through plant-mediated RNA interference
Source: Sci Rep. 2023 Apr 20;13:6477. doi: 10.1038/s41598-023-33335-4 (PMC10119085; doi:10.1038/s41598-023-33335-4)
Supplement: Supplementary file 8 — Supplementary Legends. [file 41598_2023_33335_MOESM8_ESM.docx]

**Supplementary File**

**Figure S1.** *S. sclerotiorum* lesion size and *AB3* transcript abundance of three independently transformed AT1703 lines at the T2 generation.

**Dataset S1.** GO term enrichment analysis of AT1703 and wild-type Col-0 at 2- and 3-days post inoculation.

**Dataset S2.** AT1703 dsRNA region targeting *S. sclerotiorum ABHYDROLASE-3*.

**Dataset S3.** List of primers used.

**Dataset S4.** Significantly differentially expressed gene lists in AT1703 and wild-type Col-0.

**Dataset S5.** SeqEnrich AT1703 shared transcription factor network analysis data.

**Dataset S6.** Arabidopsis count expression data.
